# The Golgi Apparatus as an Anticancer Therapeutic Target

**DOI:** 10.3390/biology13010001

**Published:** 2023-12-19

**Authors:** Marta Martins, João Vieira, Catarina Pereira-Leite, Nuno Saraiva, Ana Sofia Fernandes

**Affiliations:** 1 CBIOS—Universidade Lusófona’s Research Center for Biosciences & Health Technologies, Campo Grande 376, 1749-024 Lisboa, Portugal; marta.martins@ulusofona.pt (M.M.); joao.vieira@ulusofona.pt (J.V.); catarina.leite@ulusofona.pt (C.P.-L.); 2 Department of Biomedical Sciences, University of Alcalá, Ctra. Madrid-Barcelona Km. 33.600, Alcalá de Henares, 28871 Madrid, Spain; 3 LAQV, REQUIMTE, Departamento de Ciências Químicas, Faculdade de Farmácia, Universidade do Porto, Rua de Jorge Viterbo Ferreira 228, 4050-313 Porto, Portugal

**Keywords:** cancer, Golgi apparatus, therapy, nanotechnology, subcellular targeting

## Abstract

**Simple Summary:**

Even though scientists discovered the Golgi apparatus (GA) more than 125 years ago, there are only a few treatments that focus on this important part of the cell. The GA is a central hub in our cells, helping modify and move proteins and lipids. When the GA does not work correctly, it can affect cell processes linked to cancer. This dysregulation can impact how proteins are changed, where they go in and outside the cell, how cells use energy, or even the structure of the extracellular matrix and the environment. That is why targeting the GA could be an appealing way to treat cancer. Surprisingly, there are no anticancer drugs approved that specifically target the GA. But there is hope that new methods, like the use of nanoparticles to deliver drugs, might change that. This article looks at the drugs that are being explored for targeting the GA in cancer and the different ways scientists deliver these drugs. It also addresses the possible benefits and challenges of modulating the GA activity to treat cancer.

**Abstract:**

Although the discovery of the Golgi apparatus (GA) was made over 125 years ago, only a very limited number of therapeutic approaches have been developed to target this complex organelle. The GA serves as a modification and transport center for proteins and lipids and also has more recently emerged as an important store for some ions. The dysregulation of GA functions is implicated in many cellular processes associated with cancer and some GA proteins are indeed described as cancer biomarkers. This dysregulation can affect protein modification, localization, and secretion, but also cellular metabolism, redox status, extracellular pH, and the extracellular matrix structure. Consequently, it can directly or indirectly affect cancer progression. For these reasons, the GA is an appealing anticancer pharmacological target. Despite this, no anticancer drug specifically targeting the GA has reached the clinic and few have entered the clinical trial stage. Advances in nanodelivery approaches may help change this scenario by specifically targeting tumor cells and/or the GA through passive, active, or physical strategies. This article aims to examine the currently available anticancer GA-targeted drugs and the nanodelivery strategies explored for their administration. The potential benefits and challenges of modulating and specifically targeting the GA function in the context of cancer therapy are discussed.

## 1. Introduction

### Why Is the Golgi Apparatus a Relevant Organelle for Cancer Therapy?

The Golgi apparatus (GA) is an ancient and fundamental eukaryotic organelle found in almost all eukaryotic organisms and cell types [1]. Situated at the vital intersection of the endocytic and exocytic pathways, the GA assumes a pivotal role as a crucial transport center for eukaryotic cells. This organelle serves as a dynamic site for protein and lipid modification and acts as the key orchestrator for lipid and protein sorting and as a store for several molecules and ions [2]. Given its central involvement in membrane and protein trafficking and secretion, and in the storage capacity of second messengers (such as Ca^2+^), it comes as no surprise that dysregulation of GA function is often associated with human pathologies, such as Batten’s disease [3] or more prevalent conditions like Alzheimer’s disease [4] or cancer [5].

The GA involvement in various cancer hallmarks is of paramount importance, shaping the malignant phenotype of cancer and tumor stromal cells (reviewed in [6]). From aberrant glycosylation to enhanced secretion, the GA apparatus orchestrates a series of intricate molecular events that can contribute to shape cancer progression. It acts as a central hub for the modification and trafficking of cell surface and secreted proteins, allowing cancer cells to evade apoptosis and sustain proliferative signaling. Additionally, it can promote angiogenesis and shape the tumor microenvironment, namely by controlling extracellular matrix (ECM) composition and stromal cell behavior. By regulating the processing and maturation of critical signaling molecules, the GA can have a profound impact on the activation of key oncogenic pathways, thus driving the uncontrolled growth of cancer cells. Moreover, the GA plays a crucial role in the acquisition of invasive and metastatic capabilities by cancer cells. It modulates the synthesis and trafficking of most ECM components and of ECM-modifying enzymes such as matrix metalloproteinases (MMPs), which are crucial enzymes involved in ECM remodeling [7]. By facilitating the secretion and activation of this type of protein, the GA enables cancer cells to breach the physical barriers of the surrounding tissues [5] and initiate the metastatic cascade. Furthermore, the GA is involved in the dysregulated post-translational protein modifications, such as the glycosylation patterns, observed in cancer cells [8]. Aberrant glycosylation of proteins and lipids, catalyzed by GA-resident enzymes, leads to the generation of unique carbohydrate structures on the cell surface. These altered glycan structures play a significant role in cancer cell adhesion, migration, and immune evasion, further fueling tumor progression and metastasis [8]. The GA acts as a key regulator of these glycosylation processes, thus having a profound influence on the functional properties of cancer cells. Some less explored facets of this enigmatic organelle also have the potential to play a significant role in influencing tumor cells by impacting signal transduction, redox regulation, and ion homeostasis. By controlling the flow of several ions (Ca^2+^ and H^+^) through the secretory pathway to the extracellular space, to the cytoplasm, or to specific organelles such as the mitochondria, this organelle can affect the pH of extracellular milieu, metabolic adaptation, and reactive oxygen species (ROS) accumulation [9,10]. Thus, it may indirectly influence the proliferation, survival, and motile properties of cancer cells. In this context, the GA emerges as a potentially central player in cancer cell biology. Understanding and targeting the GA-related mechanisms underlying cancer progression-associated molecular events hold immense potential in the quest for novel cancer therapies.

Importantly, the expression levels of several Golgi-associated or resident proteins are correlated with overall survival in multiple tumors and can potentially be used in some cases as biomarkers for cancer diagnostics. This is the case for the Golgi Phosphoprotein 3 (GOLPH3) [11,12,13], the Member RAS Oncogene Family (RAB1B) [14], or the Golgi Membrane Protein 1 (GOLM1) [15], thus highlighting the potential anticancer effects that may arise from modulating such pathways.

Given the important role of the GA in defining the cancer and cancer-associated cell phenotype, regulating the processes that occur or depend on this organelle has been suggested as a potential strategy to control tumor progression [16]. One of the most explored approaches in this context is the pharmacological inhibition of the classical ER/GA secretory pathway. Despite this, other approaches that target less classical aspects of GA biology were recently described, which may prove fruitful and should be further explored. This review aims to summarize the state of the art of pharmacological anticancer approaches that directly target the GA components, as well as the nanotechnologic strategies available to deliver those GA-targeting drugs.

## 2. Pharmacological Approaches

### 2.1. Golgi Apparatus Trafficking Disrupting Agents

The GA is a pivotal element in the secretory pathway where it participates in protein and lipid synthesis, quality control, processing, and transport [2]. The dynamic nature of this organelle implies that it is continuously built by fusion of vesicles and, at the same time, it is used to produce new membrane structures. Vesicles arriving from the ER and other cellular membrane components contribute to create the GA, while, at the same time, parts of this organelle are lost to vesicle trafficking to the plasma membrane through the secretory pathway, to the ER via the retrograde movement of GA-derived vesicles, and to other destinations. The GA receives proteins from the endoplasmic reticulum (ER) and modifies them through glycosylation, phosphorylation, and proteolytic cleavage. The GA also acts as a sorting center, packaging proteins into vesicles for transport to different cellular compartments, and ensures quality control by retaining misfolded proteins for refolding or degradation. The secretory pathway, involved in protein secretion and trafficking, plays a crucial role in cancer progression. The dysregulation of this pathway affects important aspects of cancer cells, including sustained proliferative signaling, the inhibition of cell death (including apoptosis and ferroptosis), the induction of angiogenesis, and the acquisition of invasive and metastatic capabilities, and it can also modulate the amplitude and type of immune response [6]. Cancer cells often exhibit an enhanced secretion of growth factors and cytokines, promoting their own proliferation and angiogenesis and affecting immune infiltrates [17]. Additionally, the secretory pathway of tumor and stromal cells facilitates the secretion of specific elements such as enzymes that alter the structure of the ECM or of protons that directly alter the extracellular pH, thus contributing to the overall tumor microenvironment characteristics. Additionally, the changes in the trafficking of many cytoplasmic membrane proteins can affect the ability of the cell to adhere to the extracellular substratum, hence altering motility properties and potentially affecting their metastatic potential [6].

**Figure 1:**
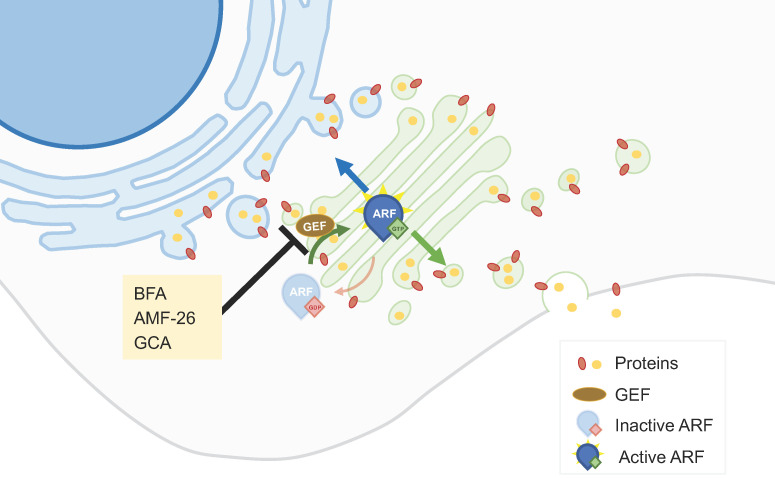
**ARF1 GEF interaction inhibitors**. ARFs can be activated by GEFs by allowing the exchange of GDP for GTP, enabling ARFs to control vesicular trafficking. Molecules like brefeldin A (BFA), AMF-26, and golgicide A (GCA) hamper the activation of ARFs and consequently affect vesicular trafficking and protein transport across the GA.

Understanding and targeting the dysregulated aspects of the secretory pathway in cancer cells may contribute to the development of effective anticancer therapies. In this context, ADP-ribosylation factor 1 (ARF1), a small GTPase protein, plays a key role in the ER-GA secretory pathway by regulating vesicle formation and cargo sorting. It controls the budding of transport vesicles from the GA, facilitating the transport of proteins and lipids to their respective destinations [18]. ARF1 can be activated by GEFs (Guanine Nucleotide Exchange Factors) by catalyzing the exchange of GDP for GTP, enabling ARF1 to carry out its functions in the secretory pathway [18]. **Brefeldin A** (BFA), a macrocyclic lactone isolated from fungi, is amongst the most studied GA-dispersing compounds. This compound hampers the interaction between members of the ARF1 guanosine triphosphatase (GTPase) family and their associated large GEFs, leading to the tubulation of early endosomes (EEs) and fusion of the GA with the ER [19] (Figure 1). BFA has shown antiproliferative and pro-apoptotic effects in different tumor cell lines [20,21]. It also decreased the invasiveness of bladder cancer cells, an effect that could be related to an increased synthesis of the glycosphingolipid GM3 [22]. BFA, and also monensin (see Section 2.2), impact the intracellular trafficking of proteins, contributing to anticancer effects. By blocking the trafficking of the multidrug resistance protein P-gp to the cell surface, this protein accumulates intracellularly. In HeLa cells, such a re-localization of P-gp increased the intracellular accumulation of daunorubicin, suggesting that this could constitute a strategy to overcome multidrug resistance [23]. Other combinatory strategies comprising BFA have been proposed. The silencing of ERGIC3 (ER-GA intermediate compartment 3, a transmembrane protein located in the ER and GA often over-expressed in cancer cells), in combination with BFA, additively inhibited lung cancer cell growth [24]. PTEN (phosphatase and tensin homolog on chromosome 10) knockout promotes GA extension and significantly sensitizes cancer cells to secretion inhibitors BFA and **golgicide A** (GCA) [25].

Although the interaction of ARF1 with ARF-GEFs is an attractive target for cancer treatment, BFA did not progress beyond the preclinical stage of drug development, due to its poor bioavailability [20]. Several other small-molecule effectors of endomembrane trafficking were previously identified through large-scale chemical genetic screens, including GCA and AMF-26 [19] (Figure 1). GCA specifically targets Golgi Brefeldin A-Resistant Guanine Nucleotide Exchange Factor 1 (GBF1), a cis-Golgi-residing ARF1-GEF [19,26]. **AMF-26**, an octahydronaphthalene derivative, is an exocytosis inhibitor that impairs the interaction between the ARF1 GTPase and its GEFs in mammals, disrupting the cis- and trans Golgi [14,15]. AMF-26 showed strong growth inhibition in several cell lines [20,27]. In addition, the oral administration of this compound induced regression of human breast cancer BSY-1 xenografts in vivo [20].

Compounds able to induce ferroptosis, a type of cell death characterized by the iron-dependent oxidative degradation of lipids, have been developed as potential anticancer agents. In addition to autophagy and apoptosis, GA-dispersing compounds like BFA, GCA, and AMF-26 can trigger ferroptosis. In multiple human cell lines, treatment with these compounds led to the accumulation of lipid peroxides, reduction in GSH levels, ROS formation, and changes in the expression levels of ferroptosis-signaling components [28].

Several protein kinases play key roles in the secretory pathway at the trans-Golgi network and are generally hyperactivated in cancer cells, contributing to cancer progression [29]. Their inhibition has thus been studied as a GA trafficking disruption strategy with potential anticancer effects. For example, **IN-9**, an antagonist of the phosphatidylinositol (PI)-4-kinase IIIβ (PI4KIIIβ), decreased cell proliferation and migration and increased apoptosis in a model of chromosome 1q-amplified lung adenocarcinoma [30]. Protein kinase D (PKD) inhibitors, such as **CID2011756** or **CRT0066101**, were shown to sensitize the drug-resistant cancer cells to platinum-based anticancer drugs [31] and to suppresses bladder cancer in vitro and in vivo [32]. Despite the importance of other kinases that are associated with the GA for the correct function of this organelle, several of these (namely, protein kinase A, protein kinase C, and Src) are also found at other subcellular localizations where they participate in other Golgi-unrelated pathways. The lack of a Golgi-restricted localization and activity in these cases means that an anticancer application that requires the specific modulation of the Golgi-localized subpopulations of any of the abovementioned kinases would be challenging.

### 2.2. Golgi-Milieu-Disrupting Agents

**Figure 2:**
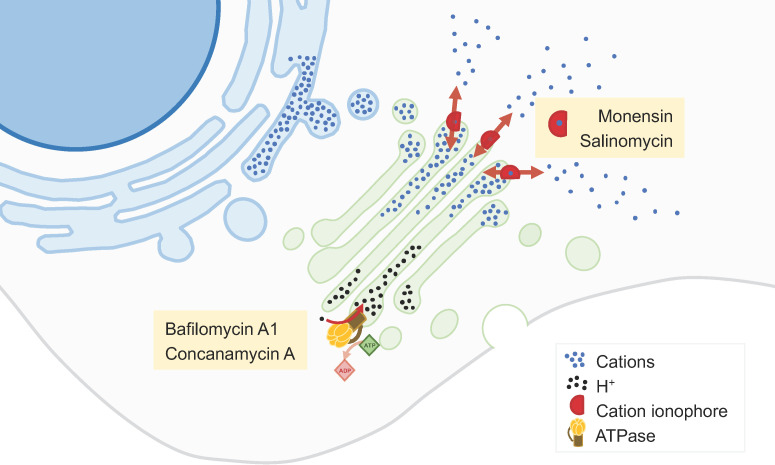
**GA cation content modulators**. The levels of various cations (namely, Ca^2+^ and H^+^) in the GA lumen are essential for its function. Molecules such as monensin andsSalinomycin act as cation ionophores, thus allowing the flow of cations across the GA membrane. The inhibition of H^+^ flow from the Golgi lumen by compounds such as bafilomycin A1 or concanamycin A leads to the alkalinization of this organelle. Changes in GA luminal cation concentrations affect various GA-associated processes like vesicular trafficking, glycosylation, and protein secretion.

**Monensin** is a polyether monocarboxylic acid obtained from fermentation using *Streptomyces cinnamonensin* that has been extensively described as a GA-disrupting agent [33,34] (Figure 2). It binds to membranes and acts as an ionophore, inducing cation imbalance and, in turn, leading to a rapid swelling of the GA and a perturbation of intracellular vesicular trafficking [33,34]. In addition, monensin alters the GA pH, affecting post-translational GA-associated processes. These alterations may trigger a GA stress response leading to apoptosis. Monensin has a potent and selective cytotoxicity to cancer cell lines with EMT-like characteristics [34].

**Salinomycin** is a monovalent cation ionophore similar to monensin, isolated from *Streptomyces albus* (Figure 2). This compound is highly selective towards cancer stem cells in many cancer types, including breast, ovarian, lung, prostate, and colorectal cancers [35,36]. Multiple mechanisms and molecular targets are described to support this activity, including mechanisms related to apoptosis, autophagy, and necrosis [35]. Among such mechanisms, salinomycin may disturb the GA, altering the expression of GA-related proteins, GA morphology, post-translational modifications of proteins, and secretion [36]. These alterations particularly affect cells that have undergone EMT and may therefore constitute a novel therapeutic approach against cancer stem cells [36].

**Prodigiosin** is a bacterial secondary metabolite that also exerts anticancer effects in different in vitro models (e.g., breast, lung, and oropharyngeal cancers), as well as in a mouse model of lung carcinoma [37]. This compound acts as an H^+^/Cl^−^ symporter, leading to an alkalization of acidic organelles such as the GA. In parallel, as demonstrated in HeLa cells, prodigiosin interferes with Golgi reassembly-stacking protein of 55 kDa (GRASP55), a protein responsible for establishing the stacked structure of the Golgi. Those two mechanisms seem to be responsible for the impairment of GA morphology, the alterations in protein functionalization and trafficking, and the effects on autophagy observed upon cell treatment with prodigiosin [38].

GA alkalinization may also be achieved by inhibition of vacuolar H^+^-ATPase (V-ATPase; Figure 2). V-ATPase is generally overexpressed in cancers and its inhibition limits the growth and metastatic properties of cancer cells [39]. V-ATPase inhibitors, such as **bafilomycin A1** or **concanamycin A**, were shown to suppress cancer cell proliferation and invasion, as well as to produce synergistic effects when combined with chemotherapy [39,40].

### 2.3. Inhibitors of the Synthetic Glycosilation Machinery

**Figure 3:**
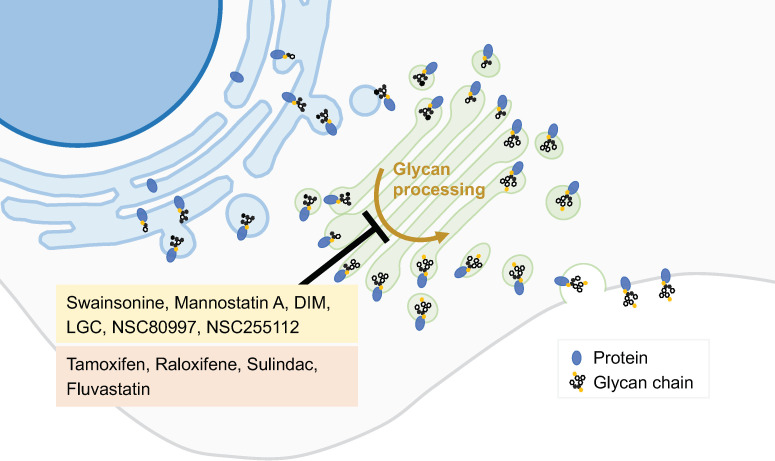
**GA-glycan-processing modulators**. Several steps of the glycosylation process occur in the GA. Modulation of this process can alter glycosylation patterns and consequently protein function. Several molecules have been initially identified as glycan-processing modulators (yellow box), and drugs used for other purposes were also reported to possess this type of activity (red box).

The inhibition of glycosidases interferes with the biosynthesis of glycans on cell surface glycoproteins and has thus been explored as an anticancer strategy of great interest. Some examples of glycosylation inhibitors studied as anticancer agents are given below. Targeting the GA α-mannosidase (GM) prevents the formation of ß1,6-branched-complex-type *N*-glycans. However, all known potent GM inhibitors also interfere with lysosomal α-mannosidase (Lman). The lack of selectivity of the inhibitors developed so far has precluded their clinical use [41]. **Swainsonine**, **mannostatin A**, and **1,4-dideoxy-1,4-imino-D-mannitol (DIM)** are naturally occurring inhibitors of α-mannosidase II that are effective in nanomolar concentrations (Figure 3). Swainsonine was developed in hydrochloride salt form, under the product code **GD0039**, which is orally available. This product showed significant in vitro and in vivo antitumor activities and beneficial immunomodulatory effects. However, in a clinical trial enrolling 17 patients with locally advanced or metastatic renal cell carcinoma, no antitumor activity was observed [41].

Eisenberg-Lerner et al. have studied the impact of inducing GA stress in multiple myeloma cells, which are characterized by a heavy load of glycoprotein production and secretion. Two pharmacological modulators were used: monensin, an ionophore that neutralizes luminal pH and blocks intra-GA trafficking described above; and **lithocholylglycine** (LCG), which inhibits 2–3 linkage sialyltransferase activity (Figure 3). Both compounds exhibit GA-stress-induced toxicity more pronounced in multiple myeloma than in a panel of other cancer cell lines. In a multiple myeloma mice model, monensin was well tolerated and provided a beneficial outcome for the splenomegaly, which is often associated with multiple myeloma [42].

Through a cell-based screening assay using a small-molecule compound library, Sorensen et al. identified two glycosylation inhibitors. The compounds **NSC80997** and **NSC255112** inhibited glycosylation steps at the GA, including those involved in the formation of *O*-glycans, *N*-glycans, and glycosaminoglycans (Figure 3). Moreover, these compounds mediate the reversible fragmentation of the GA system. Unlike the GA-disrupting agent BFA, NSC80997 and NSC255112 do not affect the transport and secretion of glycoproteins. These inhibitors may be used to manipulate glycosylation in cancer cells, inducing the expression of truncated *O*-glycans and augmenting the binding of cancer-specific Tn-glycoprotein antibodies, thus enhancing the efficacy of immunotherapies [43].

### 2.4. Inhibitors of Peripheral Golgi-Associated Proteins

**Figure 4:**
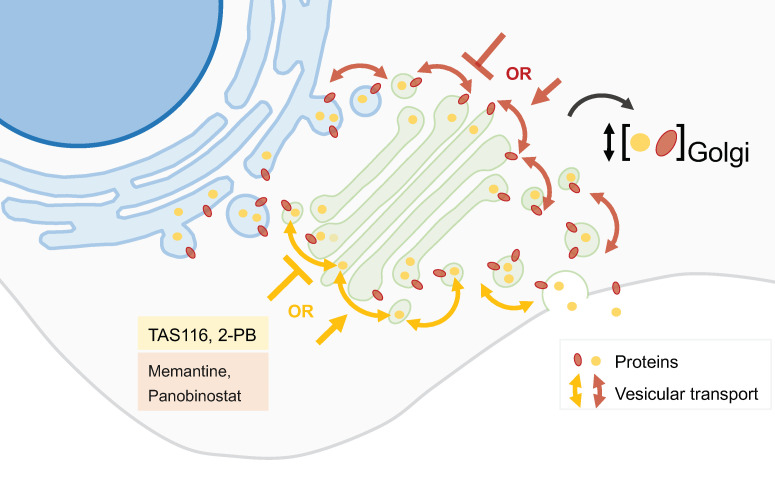
**GA protein localization modulators.** The precise location of proteins within the GA is essential for the correct function of this organelle. Some compounds can alter the location or change the concentration of specific GA proteins. Several molecules have been initially identified as capable of altering GA protein location (yellow box) while drugs used for other purposes also possess this action (red box).

Many gastrointestinal stromal tumors (GISTs) are caused by gain-of-function mutations in the Kit receptor tyrosine kinase. Imatinib improves the prognosis of GIST patients, but often becomes ineffective with prolonged use because of secondary mutations in the Kit kinase domain. Mutant Kit accumulates predominantly on the GA, whereas normal Kit localizes to the plasma membrane [44,45]. Heat-shock protein 90 (HSP90) is required for the appropriate folding of the KIT protein. **TAS-116**, an orally active HSP90 inhibitor, decreased GA-localized KIT in both imatinib-naïve and imatinib-resistant GIST cells (Figure 4). This compound inhibited the growth and induced the apoptosis of GIST cell lines with KIT activation and caused tumor growth inhibition in xenograft mouse models of imatinib-resistant GISTs. TAS-116 was also effective against gefitinib-naïve and gefitinib-resistant epidermal growth factor receptor (EGFR)-mutated lung cancer. TAS-116 was thus suggested as a novel drug to overcome tyrosine kinase inhibitor resistance in both GIST and EGFR-mutated lung cancer [45].

Other anticancer agents acting on GA are the **2-(substituted phenyl)-benzimidazole** (2-PB) compounds (Figure 4). These agents displace resident GA proteins from the juxtanuclear region, resulting in their degradation. 2-PB compounds have been shown to decrease proliferation in a variety of cell lines and suppress tumor growth in xenograft models of human melanoma, breast carcinoma, and renal carcinoma [46].

### 2.5. Drug Repurposing

Drug repurposing, i.e., the identification of clinically approved drugs with known safety and pharmacokinetic profiles for new indications, has emerged as a useful approach for the search for novel cancer treatments. Such a strategy has also been applied to GA-targeted drugs. For example, drug repositioning plays an important role in identifying potential inhibitors of GA mannosidases. A cell-based screen of a compound library identified three approved drugs, **tamoxifen**, **raloxifene**, and **sulindac**, as potential GA mannosidases inhibitors that induced accumulation of mannose-type *N*-glycan in HeLa cells [47] (Figure 3). In addition, the approved drug **fluvastatin** inhibits the mevalonate pathway and *N*-glycosylation at both the ER and GA (Figure 3). In a mouse model of post-surgical metastatic breast cancer, adjuvant treatment with fluvastatin decreased metastasis and improved overall survival [48]. Besides fluvastatin, another inhibitor of 3-hydroxy-3-methyl-glutaryl-CoA reductase (HMGCR), **pitavastatin**, has shown tumor growth inhibition in vitro, in ovo, and in mouse models of oral and esophageal squamous cell carcinoma. Its anticancer activity is likely related to the inhibition of the MET signaling pathway due to the prevention of MET maturation through dysfunction of the GA. The combination of pitavastatin with capmatinib, an MET-specific inhibitor, enhanced the tumor growth inhibitor in oral and esophageal cancers models [49].

**Memantine** is a non-competitive *N*-methyl-D-aspartate (NMDA) receptor antagonist used in Alzheimer’s disease, which has been suggested as a potential anticancer drug (Figure 4). While its tumor-suppressive effect has been attributed to the blockage of the NMDA-receptor, another mechanism was recently proposed involving the Golgi glycoprotein 1 (GLG1), an intracellular fibroblast growth factor receptor (FGFR). Memantine treatment induces the expression of GLG1 and the generation of additional truncated variants that may suppress the FGFR pathway. It also leads to variations in the intracellular distribution of GLG1. These mechanisms seem to play a crucial role in the suppression of cancer cell growth, as demonstrated in glioma and breast cancer cell models [50].

Using an image-based screening platform, Gendarme et al. identified 12 novel GA-fragmentation-inducing agents, essentially belonging to two classes of compounds: histone deacetylase inhibitors (HDACi) and DNA-damaging agents. **Panobinostat**, an HDACi used in the treatment of multiple myeloma, increases the protein trafficking rate without negatively affecting glycosylation or GA dynamics (Figure 4). Moreover, Panobinostat and (+)-JQ1, a small molecule which inhibits the bromodomain and extraterminal domain (BET) family of proteins, synergistically disperse the GA and decrease the viability in a panel of cancer cell lines [51].

## 3. Nanotechnological Approaches

Nanodelivery systems can offer additional therapeutic advantages by selectively transporting drugs to tumor cells due to passive, active, and physical targeting. These strategies may allow improved bioavailability and specificity, low-dose administration, and the decreased occurrence of side-effects. Various studies have been reported concerning the nanoencapsulation of drugs targeting GA-related processes with anticancer potential, which will be further detailed according to the type of targeting strategy employed. Moreover, the field of organelle-targeted nanodelivery systems is growing, with some studies pointing to the possibility of specifically targeting the GA [33]. The state of the art of this organelle-specific approach is also discussed in the next subsections.

### 3.1. Passive Targeting

Passive targeting to tackle cancer cells is based on the intrinsic physicochemical properties of the nanocarriers (size and surface chemistry) and the specific features of the tumor microenvironment, including the enhanced permeability and retention effect. This phenomenon leads to the accumulation of nanocarriers within tumor tissues, thanks to the upregulated angiogenesis that results in an impaired vasculature [52]. Passive targeting strategies have been investigated for the delivery of a wide range of drugs, including those with a primary mechanism of action centered in the GA, such as BFA, monensin, and salinomycin and prodigiosin.

Polymeric nanocarriers have been explored to upgrade the delivery of BFA [16,53], not only because it displays poor bioavailability [20] but also to better target the tumor tissues. Liu et al. [53] developed polyethylene glycol (PEG)–PLLA electrospun fibers able to control the release of BFA. Using human liver carcinoma HepG2 cells, the antitumor activity of these nanofibers was also demonstrated in a BFA concentration-dependent manner. This study suggests that polymer nanofibers may be a valuable nanocarrier for BFA to overcome its poor solubility and short half-life [53]. poly(DL-lactide-co-glycolide) (PLGA)-PEG nanoparticles co-loaded with BFA and the cyclooxygenase-2 inhibitor celecoxib inhibit breast cancer growth and suppress metastasis. In murine metastatic breast cancer 4T1 cells, these nanoparticles damaged the GA, induced cytotoxicity, decreased cell migration and invasion, and reduced the expression and secretion of MMP-9 and vascular endothelial growth factor [16].

Liposomes and polymeric nanoparticles were explored to deliver monensin to cancer cells so that this cation ionophore could potentiate the anticancer activity of ricin-based immunotoxins [54,55,56]. Initially, liposomes were explored and, later, PLGA (50:50) nanoparticles were developed to avoid the long-term storage issues of liposomes (oxidation and hydrolysis of phospholipids). Using HL-60 (promyelocytic) and HT-29 (colon adenocarcinoma) human tumor cell lines, both liposomes and PLGA nanoparticles caused a 40–50-fold increase in the activity of ricin immunotoxins [55]. Afterwards, PEG/PLGA diblock copolymers were used to prepare nanoparticles to load monensin with long-circulating properties [56]. These nanocarriers were found to successfully boost the in vitro cytotoxicity of anti-My9 (ricin-based immunotoxin) and of anticancer drugs like doxorubicin and tamoxifen in various tumor cell lines (sensitive and resistant HL-60, MCF-7, and SW-620). Moreover, these nanoparticles allowed the prolonged circulation of monensin during 24 h after their intravenous administration to male Sprague-Dawley rats, whereas the circulation lasted only 4 h when administering a monensin solution. This shows that the inclusion of PEG in the nanocarriers was a successful approach to provide long-circulating properties [56].

The poor aqueous solubility of salinomycin and its remarkable potency to kill cancer stem-like cells (CSCs) triggered the development of various nanosystems to encapsulate it and to improve salinomycin biodistribution and bioavailability, namely, polymeric micelles, nanofibers and nanoparticles, pegylated liposomes, and nanoemulsions [57,58,59,60,61,62]. Passive-targeting polymeric micelles made of PEG-b-poly(ε-caprolactone) (PEG-b-PCL) to load salinomycin were developed and combined with octreotide-modified paclitaxel-loaded PEG-b-PCL micelles as an alternative approach to improve the treatment of breast cancer. This dual-acting strategy was effective considering both in vitro cytotoxicity studies using the MCF-7 cell line and in vivo studies using MCF-7 xenografts [61]. Polymeric nanoparticles and nanoemulsions were also explored as platforms for combination therapy of salinomycin with conventional anticancer drugs for breast cancer treatment [57,58,59]. PLGA/D-alpha-tocopherol-polyethylene glycol 1000 succinate (TPGS) nanoparticles were developed, making use of the pore-forming and P-glycoprotein-inhibitory properties of TPGS, to co-deliver docetaxel and salinomycin for breast cancer treatment. Both in vitro and in vivo studies showed that this synergistic strategy is promising to inhibit the proliferation of breast cancer cells and breast CSCs [58]. A similar strategy combining salinomycin and doxorubicin in a Poly(lactic) acid (PLA)-based hybrid block copolymer was also effective against chemo-resistant cancer cells and CSCs derived from cancer patients. Moreover, these dual-acting nanoparticles were tested in a mice model of breast cancer, inducing a remarkable tumor regression and inhibiting tumor recurrence [59]. In vitro studies on the impact of nanoemulsions co-loading paclitaxel and salinomycin on stem cell-enriched mammospheres also revealed the potential of this synergistic approach to improve anticancer efficacy and eventually reduce systemic toxicity [57]. Nanosystems loading salinomycin were also designed to potentially treat glioblastoma and colorectal cancer [60,62]. Considering glioblastoma, salinomycin-loaded PLGA nanofibers were developed for local therapy and recurrence prevention upon implantation in the brain cavity after surgical removal of the tumor. Using human glioblastoma U-251 cells, this nanosystem improved cytotoxicity induction via apoptosis in comparison with free salinomycin by inducing intracellular ROS and upregulating the expression of RB Transcriptional Corepressor Like 1 and 2 tumor suppressor genes and of caspase 3 [60]. In vitro cytotoxicity studies on SW-620 human colorectal cancer cells showed that PEGylated liposomes co-encapsulating gemcitabine and salinomycin are more potent than the conventional drug alone (encapsulated or free), thus boding well for the use of this strategy also for the treatment of colorectal cancer [62].

Recently, chitosan-based nanoparticles were developed for the delivery of prodigiosin to ultimately improve the bioavailability of this bacterial pigment. The encapsulation of prodigiosin slightly improved the anticancer activity towards A-549 lung carcinoma cells. Moreover, a promising safety profile of the nanodelivery system was demonstrated using in vitro and in vivo (*Daphnia magna* and zebrafish) toxicity assays [63]. A different strategy was reported by Gugu et al. [64] using lipid nanoparticles to load prodigiosin for parenteral administration to eventually treat triple-negative breast, lung, and colon cancers.

### 3.2. Active Targeting

**Figure 5:**
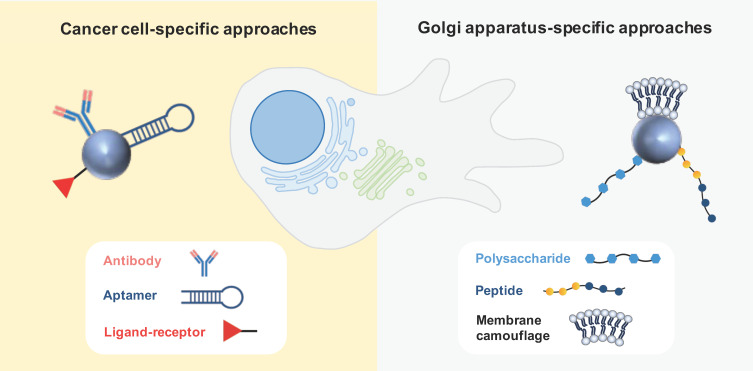
**Active strategies for drug nanodelivery targeting cancer cells or GA**. Antibodies, aptamers, and ligand–receptor interactions have been explored as cancer-cell-specific approaches, whereas polysaccharides (chondroitin sulfate), peptides, and membrane camouflage have been investigated as GA-specific approaches.

Active targeting is based on ligand-conjugated nanosystems that interact with molecules expressed at the surface or specific organelles of tumor cells. Antibodies and aptamers are widely recognized as highly specific targeting moieties. Other active strategies often explored in the field of anticancer therapy comprise ligand–receptor interactions and peptides [52]. Various active-targeting approaches have been used to specifically deliver GA-acting drugs to cancer cells or to the specific organelle (Figure 5).

#### 3.2.1. Cancer Cell-Specific Approaches

Active targeting using antibodies was considered for the delivery of monensin and salinomycin to tumor cells. Singh et al. [54] developed monensin-loaded liposomes functionalized with monoclonal antibodies (mAbs) targeting the murine anticarcinoembryonic antigen (CEA), which is well known to be overexpressed in cancer cells. In vitro experiments revealed that mAb-targeted monensin liposomes exhibited a potency 100 times greater than that of monensin liposomes, significantly enhancing the activity of ricin immunotoxins against diverse tumor cell lines. To specifically target human epidermal growth factor receptor 2 (HER2)-positive breast cancer cells, anti-HER2 nanoparticles were developed to deliver salinomycin. First, PLGA nanoparticles decorated with an anti-HER2 antibody were engineered, showcasing their specific uptake by HER2-positive breast cancer cells (MCF-7) while enabling controlled drug delivery [65]. Afterwards, Li at al. [66] developed anti-HER2 polymer–lipid hybrid nanoparticles. In this case, both in vitro and in vivo studies supported the use of antibody-targeting strategies to enhance cytotoxic effects, inhibit tumor growth, and reduce the tumorsphere formation efficiency in comparison with non-targeting nanoparticles and free drugs.

Aptamers were also considered as an active targeting strategy for the nanodelivery of salinomycin to treat melanoma and osteosarcoma. The salinomycin therapeutic efficacy against CSCs was the rationale to develop lipid-polymer nanoparticles functionalized with anti-CD20 aptamers, as CD20-positive stem cells are key for the development and metastatic behavior of melanoma. The selectivity of these nanoparticles was demonstrated and they were more cytotoxic than the non-decorated ones. Considering melanoma xenografts in mice, tumor growth inhibition was superior when administering aptamer-targeting nanoparticles compared to non-targeting nanoparticles or free salinomycin, showing the potential of aptamers as an active targeting approach [67]. Chen et al. [68] proposed dual-active targeting to deliver salinomycin-loaded lipid-polymer nanoparticles to osteosarcoma cells and CSCs. For that, the nanocarriers were labeled with CD133 and EGFR aptamers to selectively target CD133-positive osteosarcoma CSCs and EGFR-overexpressing osteosarcoma cells, respectively. In vitro and in vivo studies in mice showed the therapeutic superiority of the dual-targeting strategy in comparison to single- or non-targeting nanoparticles.

An active targeting approach based on the ligand–receptor interaction was proposed by Zhu et al. [69] by designing salinomycin-encapsulated 1,2-Distearoyl-sn-glycero-3-phosphorylethanolamine (DSPE)-PEG-methotrexate nanomicelles to target head and neck squamous cell carcinoma (HNSCC) cells and CSCs. In this approach, methotrexate was used as a cytotoxic agent and a homing ligand, due to its structural similarity with folic acid, thereby displaying an affinity to the overexpressed folate receptors in cancer cells. Beyond in vitro inhibitory benefits, an effective suppression of tumor growth was observed in nude mice bearing an HNSCC xenograft by combining salinomycin and methotrexate in this nanoplatform.

#### 3.2.2. Golgi-Apparatus-Specific Approaches

The central involvement of the GA in orchestrating signaling pathways governing migration, invasion, and angiogenesis has captivated the interest of various research groups, leading to the development of GA-specific active targeting nanoparticles based on ligand–receptor interactions, peptides, and membrane camouflage. These nanosystems are intended to disrupt both the structural characteristics and functional aspects of the GA that underlie metastatic processes in cancer.

Chondroitin sulfate (CS)—a biocompatible, non-immunogenic polysaccharide—was described as an interesting antitumor vector due to its affinity for the surface receptor CD44, which mediates the uptake by tumor cells. Interestingly, this polysaccharide was also found to accumulate in the GA, leading to the design of nanocarriers to selectively target the GA for cancer treatment [70,71]. This strategy was employed to co-deliver a chemotherapeutic agent (doxorubicin or paclitaxel) along with retinoic acid (RA). This derivative of vitamin A was chosen because it was observed to induce changes in the morphology of the GA. The expectation was that this combination would lead to a synergistic effect in treating cancer. In fact, a prodrug nanoparticle composed of RA-conjugated CS and loading paclitaxel showed promising in vitro efficacy in terms of inhibiting cancer cell migration and invasion, and angiogenesis. Furthermore, this nanoformulation exhibited in vivo benefits, including the suppression of tumor growth and the prevention of metastasis in mice carrying metastatic 4T1-Luc tumors [70]. A similar approach was investigated by Luo et al. [71] to fight liver cancer. These authors reported that CS-decorated lipid nanovesicles co-loading doxorubicin and RA were uptaken by SMMC-7721 hepatoma cells and hepatic stellate cells. Moreover, the nanosystems accumulated in the GA, causing structural changes and resulting in ECM biosynthesis inhibition. In vivo studies also demonstrated the benefit of the nanosystem loading both drugs in terms of tumor penetration, anticancer efficacy, and therapeutic safety. Although the data reported thus far are quite promising, there remains an important question regarding whether the observed disruption in GA structure is a direct consequence of the nanoparticle-GA interaction or if it is an indirect outcome of drug-induced cell death.

Li et al. [72] developed an elegant nanomechanical strategy, based on a transformable peptide, to selectively disrupt the GA structure to avoid drug resistance in cancer therapy. The proposed peptide forms nontoxic nanoparticles in aqueous medium but it is activated by furin cleavage in the GA, causing the in situ formation of left-handed helical fibrils. These structures decreased the viability of MCF-7 and A549 cancer cell lines and reduced the tumor size in MCF-7-tumor-bearing mice [72].

Membrane camouflage is another ingenious approach to actively target the GA of tumor cells. This strategy was recently explored to suppress cancer metastasis and to improve immunotherapy [73,74]. Chen et al. [73] used the membrane from B16-F10 melanoma cells to coat PLGA nanoparticles to ultimately evade lysosomal degradation through caveolae-associated endocytosis and selectively accumulate within the ER-Golgi network, facilitated by the presence of SNARE proteins on its surface. This nanosystem was employed at first to load BFA and then to co-load JQ1, a PD-L1 inhibitor, showing a synergistic effect promoting antitumor immunity and reversing immunosuppression. In in vivo experiments with B16-F10-tumor-bearing mice, the homologous cancer cell membrane coating exhibited superior and sustained antitumor efficacy when compared to PEGylated and red blood cell membrane (RBC) coatings. To target the whole cascade of metastasis, a similar approach was attempted by using a hybrid erythrocyte and tumor cell membrane (4T1) coating on PLGA nanoparticles (Hyb-NP) to load monensin. This membrane camouflage strategy was designed not only to increase circulation time but also to recognize primary, circulating, and colonized tumors. Due to the inhibition of the exocytosis of Hyb-NP from the GA by monensin, the nanocarriers were accumulated in this subcellular compartment, functioning as a reservoir. This approach facilitated the inhibition of metastasis initiation, suppressed the release of metastasis-related cytokines, and hindered directional cell migration. Using an orthotopic and spontaneous metastatic breast tumor model in mice, Hyb-NP-loading monensin almost eradicated the metastatic tumor, with a significantly higher antitumor activity than RBC-coated nanoparticles and free drugs [74].

### 3.3. Physical Targeting

Physical targeting encompasses stimulus-responsive nanosystems that release the cargo upon the effect of an endogenous (pH, hydrogen peroxide concentration, redox state, and hypoxia) or exogenous (light, magnetic fields, heat, and ultrasonic radiation) stimulus [75].

An interesting strategy proposed is a smart nanosystem that takes advantage of the acidic microenvironment of the GA. Bovine serum albumin (BSA) nanoparticles containing a pH-responsive photothermal ablation agent (pH-PTT) based on cyanine dyes for photothermal therapy (PTT) were developed to ultimately kill cancer cells through hyperthermia upon incidence of light. These nanosystems preferentially accumulated in the GA of cancer cells compared to normal cells, and thus can be specifically activated by the acidic environment of the GA in cancer cells. This resulted in a higher photothermal toxicity towards cancer cells, as observed in vitro. In addition, a remarkable photothermal anticancer effect was observed in HePG-2-tumor-bearing mice [76].

A multi-targeting approach was developed by Xu et al. [77] for designing a gene delivery system to fight drug-resistant cancer. In this nanocomposite, low-molecular-weight polyethyleneimine (LMW-PEI) was conjugated with β-cyclodextrin to form the polyplex core. This core was further coated by poly-γ-glutamic acid (γ-PGA) or arginylglycylaspartic acid (RGD)-modified γ-PGA which can actively target tumor-associated receptors, namely, gamma-glutamyl transpeptidase and integrin (respectively). Moreover, two stimulus-responsive agents are present in the nanocomposite: the detachment of γ-PGA from the surface coating is activated by the tumor acidic micro-environment and the disulfide cross-linking between LMW-PEI and β-cyclodextrin is disassembled in a redox-responsive manner, favoring the cargo delivery. In this study, the nanocomposite was engineered for the co-delivery of TNF-related apoptosis-inducing ligand (TRAIL) DNA and monensin. It exhibited significant efficacy in halting the growth of chemoresistant tumors, especially when RGD-modified γ-PGA was employed as the targeting agent. These findings indicate that the pTRAIL/monensin combination could serve as a promising second-line therapy for patients with chemoresistance.

## 4. Conclusions and Future Perspectives

Several GA proteins have been pointed out as cancer biomarkers, including GP73, GOLPH2, GOLPH3, GOLM1 [11,12,13,78,79,80], and α-mannosidase 1A (MAN1A1) [81,82]. Despite this and considering the involvement of the GA in a great number of cancer initiation and progression processes, most of the therapeutic strategies involving this organelle have failed to translate into clinical practice. Manipulating GA-associated events to control the fate of cancer cells presents a major challenge, primarily because it may be necessary to target specific processes within the organelle without inadvertently affecting others that could result in undesirable effects. Also, the full spectrum of repercussions that arise from the dysregulation of GA-specific processes in cancer cells is still poorly understood. Besides the GA proteins described in this review, other processes that involve GA-associated proteins could be targets of anticancer drugs. This is the case of NRLP3 inflammasome or GOLPH3-dependent processes [83].

While various anticancer approaches targeting the GA have been proposed, no compound has advanced to clinical practice thus far. In fact, there is an evident scarcity of clinical trials focused on GA-targeting agents. The absence of well-defined mechanisms of action for these molecules may largely contribute to this lack of progress. The growing understanding of GA biology along with technological advances may allow the design of optimized anticancer molecules acting on this organelle. Recently, a phase IA/IB study evaluating TAS-116 in patients with advanced solid tumors reached completion (NCT02965885). Additional clinical trials focused on this drug are in the recruitment phase, as listed on clinicaltrials.gov, accessed on 4 December 2023. An additional source of optimism lies in the realm of drug-repurposing strategies. Leveraging the established safety profiles of existing drugs may expedite their translation into clinical practice.

Several strategies involving targeting DNA-damage-inducing agents (such as platinum complexes or doxorubicin) to the GA have shown some interesting anticancer activity [70,71,84,85]. Despite this, it is not yet clear if these compounds can significantly trigger processes directly at the GA or if they act mainly via their DNA damage activity. It would be important to clarify if the cellular effects arising from these molecules that typically include GA fragmentation and loss of GA functions such as vesicle trafficking and glycosylation, are indeed GA-dependent or are mostly a downstream consequence of cell death.

The simultaneous involvement of this organelle in numerous cell biology processes makes it difficult to modulate one function without disturbing several others. Additionally, targeting molecules to the GA compartments is a great challenge and inefficient and nonspecific intracellular targeting increases the likelihood of side-effects. To specifically target one GA process without affecting all others, an integrated understanding of cancer-specific dysregulated GA processes and elements and their specific contribution to cancer hallmarks is necessary. This degree of complexity arises from the diverse array of roles performed by the GA, emphasizing the need for precise and selective intervention strategies to achieve desired therapeutic outcomes. In this context, nanotechnology may eventually be a solution to precisely target the GA.

Various nanotechnological approaches have been proposed for the delivery of GA-process-modulating drugs to cancer cells, making use of passive, active, and physical targeting strategies with successful outcomes in cellular and animal studies. In the last 4 years, elegant targeting approaches, making use of ligand–receptor interactions, transformable peptides, and membrane camouflage, have been suggested for the specific delivery of anticancer compounds to the GA. This may have only been the beginning of the field of organelle-specific targeting, which may be an asset to specifically modulate the GA functions with benefits in terms of anticancer efficacy, particularly related to the control of metastasis and chemoresistance. Thus, it is expected that some of these approaches may reach clinical trials in the near-future. Despite the results of the foreseen trials, questions remain regarding the potential translation of these nanoplatforms into clinical practice as these advanced delivery systems are associated with laborious and costly production methods. Nevertheless, the journey to develop a cost-effective, clinical-translatable, GA-targeting nanoplatform with remarkable anticancer efficacy and proper safety profiles is ongoing with promising initial breakthroughs.

## Data Availability

Not applicable.
